# Forced into shape: Mechanical forces in *Drosophila* development and homeostasis

**DOI:** 10.1016/j.semcdb.2021.05.026

**Published:** 2021-12

**Authors:** Giulia Paci, Yanlan Mao

**Affiliations:** a MRC Laboratory for Molecular Cell Biology, University College London, Gower Street, London WC1E 6BT, UK; b Institute for the Physics of Living Systems, University College London, Gower Street, London WC1E 6BT, UK

**Keywords:** Mechanical forces, *Drosophila* development, Morphogenesis, Patterning, Homeostasis

## Abstract

Mechanical forces play a central role in shaping tissues during development and maintaining epithelial integrity in homeostasis. In this review, we discuss the roles of mechanical forces in *Drosophila* development and homeostasis, starting from the interplay of mechanics with cell growth and division. We then discuss several examples of morphogenetic processes where complex 3D structures are shaped by mechanical forces, followed by a closer look at patterning processes. We also review the role of forces in homeostatic processes, including cell elimination and wound healing. Finally, we look at the interplay of mechanics and developmental robustness and discuss open questions in the field, as well as novel approaches that will help tackle them in the future.

## 1 Introduction

Decades of research have shaped our understanding of how genetic and biochemical programs guide morphogenesis of tissues with specific patterns [1]. However, we do not fully understand the interplay of these genetic programs with intrinsic and extrinsic mechanical forces to give rise to functional three-dimensional tissue shapes with distinct physical properties. Recent efforts that leverage on interdisciplinary approaches combining developmental biology, physics and engineering are starting to shed light on the links between mechanical forces, gene expression and signaling in morphogenesis and homeostasis of tissues.

In this article, we aim to provide a broad overview to highlight the multiple roles of mechanical forces in *Drosophila* development and homeostasis [2], looking at how they can create and maintain tissue shapes while also enabling tissues to respond to environmental stimuli and keep their integrity. The overall review and the individual sections are organized to follow developmental timing. Firstly, we will discuss the interplay between mechanics and tissue growth during development, both in terms of division rate and division orientation. We will then cover key examples of morphogenetic processes where mechanical forces play a key role in sculpting 3D tissue shapes, including tissue elongation, folding and tubulogenesis. We will further summarize the role forces play in the patterning and refinement of tissue organization, as well as homeostatic processes including cell competition and wound healing. Finally, we will discuss recent examples of processes where tissue mechanical properties have been found to play a role in conferring developmental robustness. We will conclude with an outlook on future directions, both in terms of unexplored research directions and novel methods and technologies that will be crucial to further our understanding of developmental forces *in vivo*.

## 2 Mechanics of growth control during *Drosophila* development

### 2.1 Mechanical feedbacks in cell growth and division rate

**Fig. 1:**
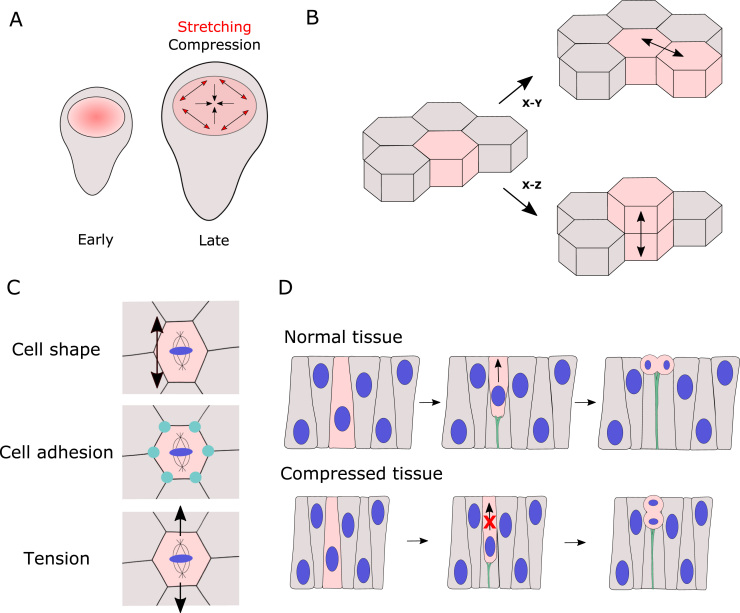
Mechanics and cell division. (A) A gradient of proliferation rates in early *Drosophila* wing disc pouch contributes to a global tension pattern that promotes proliferation at the periphery and suppresses it at the center. (B) Epithelial cells can divide in the plane of the tissue (X-Y) or in the apico-basal direction (X-Z), giving rise to additional cell layers. (C) Several factors influence the orientation of cell divisions, including cell shape, molecular machinery enriched at cellular junctions and mechanical tension. (D) In pseudo-stratified epithelia, crowding caused by mechanical compression can impair nuclear migration and prevent a correct mitotic nuclear positioning.

In *Drosophila,* the most studied system for organ growth is the wing imaginal disc, which grows from approximately 50 cells to 50,000 during the larval stages of development and has been shown to scale in size with animal body size under starvation conditions [3]. Growth factors, including Decapentaplegic (Dpp), control wing growth: Dpp is produced locally by a stripe of cells and gradually spreads out, forming a morphogen gradient [4], [5]. Despite this sustained morphogen gradient, however, proliferation of cells occurs roughly uniformly throughout the entire tissue, especially towards the end of wing disc growth. Theoretical models have suggested that mechanical feedback could act as a regulator of growth in such a scenario, with local growth rates modulated by mechanical stress [6]. In this way, a patch of cells growing faster than the surrounding tissue would experience compression and as a response would downregulate its growth rate to reduce mechanical stress (Fig. 1A). This model is consistent with several observations that mechanical forces can indeed regulate cell proliferation in cell culture, with compression inhibiting growth and stretch promoting it [7], [8], [9]. Mechanical feedback of growth has also been suggested to act as a size-control mechanism [10], [11], whereby a growing tissue would experience an increasing compressive stress due to external constraints (e.g. due to the extracellular matrix [ECM] or neighboring tissues) that would, in turn, slow down growth to reach the final tissue target size.

What are the molecular mechanisms behind this mechanical feedback of growth? The Hippo pathway is a key regulator of physiological and oncogenic growth in several species [12], [13] and has emerged in recent years as the potential connection between mechanical stress and growth [14]. A core element of the pathway is the kinase Warts (Wts), that phosphorylates and negatively regulates the transcriptional co-activator Yorkie (Yki, named YAP or TAZ in mammals), leading to transcriptional downregulation of genes involved in growth and apoptosis, including Cyclin E and DIAP1 [15], [16]. Several models have been proposed for how Yki responds to mechanical forces in *Drosophila*. In wing discs, Yki activity has been shown to respond to changes in cytoskeletal tension, through the co-recruitment of the Ajuba LIM protein (Jub) and Wts to adherens junctions under conditions of high tension [17]. Changes in Yki activity and corresponding Jub pathway components have also recently been reported during the normal development of wing discs [18]. It has also been shown that cytoskeletal regulators Zyxin and Enabled can regulate Yki-dependent organ growth via regulation of Hippo pathway component Expanded [19].

Finally, it is important to note that different mechanisms can enable developing tissues to control their growth rate and final size, even within the same organism. For instance, a recent study focusing on the *Drosophila* abdominal epidermis found that growth arrest was independent of changes in apical area and tension [20]. In this tissue, growth termination appears to occur by the rapid stochastic switching of cell populations to proliferation arrest, and may also require remodeling of the basal ECM [20]. Therefore, different mechanical cues in the form of cell crowding, tension and interactions with the ECM can provide alternative growth termination strategies that enable tissues to robustly achieve their target size.

### 2.2 Mechanics and cell division orientation

During development, the orientation of cell division in the plane of the epithelium (X-Y, Fig. 1B) is another factor that can dramatically impact the final tissue shape. In the wing disc, cells have been shown to divide preferentially along the proximal-distal (PD) axis, giving rise to an elongated tissue [21]. Control of this process depends on the Fat/Dachsous polarity pathway, through the polarization of the unconventional myosin Dachs that induces a polarized apical constriction and thus orients cell divisions along the elongated PD axis [21], [22], [23]. Cells located in proximal regions of the wing pouch, on the other hand, divide tangentially [24], [25], despite the same radial (PD) polarized pattern of Dachs expression. The reason for this orientation is a global pattern of mechanical stress present in the wing disc, whereby cells in the proximal regions of the pouch are stretched tangentially and have a higher junctional tension compared to cells located in the center of the pouch [24], [25].

What is the source of this global mechanical stress pattern? A potential explanation is growth inhomogeneity: quantification of proliferation rates in the wing disc revealed that, at early stages of larval development (48–72 h AEL), proliferation is faster in the center of the pouch compared to proximal regions [25]. This transient growth rate differential has been shown to be sufficient for the generation of the observed global tension pattern, thus driving tissue shaping and growth via tension-dependent oriented cell divisions [25] (Fig. 1A). A further supporting evidence of this self-regulated growth mechanism is that genetically perturbing the feedback between growth and mechanics results in aberrant patterns of mechanical strain and proliferation [26]. More recently, cell intercalations (neighbor exchanges) have also been implicated in the formation and maintenance of the global stress pattern [27], [28]. While previous observations had reported few or unoriented intercalations in the wing pouch [29], [30], quantification of cell movements in wing disc cultured using improved conditions found frequent radially patterned T1 transitions [27], [28]. This explains an apparent conundrum of how tangential cell elongation is maintained in the wing disc, despite homogenizing of growth differentials after the mid-third instar stage (~80 h AEL onwards). It is proposed that these radial cell neighbor exchanges are active processes, and account for the patterns of cell elongation independently of sustained differential growth and PCP pathways. Instead, it is suggested that cell shape and tissue tension patterns are sustained through self-organization via ﻿a mechanosensitive feedback that requires Myosin IV activity [28].

The molecular mechanisms controlling division orientation have been investigated since early observation in the late 1800 s that cells preferentially divide along their longest axis (the so-called “﻿Hertwig’s rule”), see [31], [32] for recent reviews on the topic. The canonical molecular machinery involved in shape sensing and spindle orientation comprises the dynein-associated protein Mud, which is enriched at tricellular junctions (TCJs) and retains its interphase cortical distribution during mitosis [33], [34]. Despite it being highly conserved, however, this molecular machinery is not universal, and different tissues employ different mechanisms to control division orientation (Fig. 1C). For example, spindle orientation in the *Drosophila* follicular epithelium and ﻿early embryonic ectoderm has been shown to be independent of cell shape [35], [36] and rather depend on tissue-level tension. Finally, a recent study in the fly notum, where cells from different regions have similar shape but different levels of tension, found that isotropic tissue tension is important to enable the spindle to orient with the long cell axis [37].

The above mechanisms orienting divisions can only function correctly if the cells are able to accurately divide in the X-Y plane of the epithelium (as opposed to in the X-Z apical/basal axis, Fig. 1B). If alignment of the spindle to the epithelial plane is defective, cells can delaminate and undergo apoptosis, potentially compromising epithelial integrity [38]. Several molecular factors have been shown to control this [39], and even within the same epithelium, division orientation in X-Y and X-Z can be controlled by different mechanisms [35]. The mechanical properties of tissues can also influence the accuracy of planar cell divisions, especially in pseudo-stratified epithelia, where mitotic nuclei need to translocate to the apical side in order for rounding and planar-oriented divisions to occur [40]. A recent study in *Drosophila* wing discs, for example, found that nuclear movement strongly depends on nuclear density: if density is increased through mechanical compression, nuclear migration is perturbed, preventing a correct mitotic nuclear positioning [41], Fig. 1D. Consistently, mitotic nuclear dynamics change during development as cell density increases, corresponding to an increasing requirement for the formin Diaphanous to achieve correct apical nuclear positioning [41] and planar cell division.

In summary, oriented cell divisions can shape developing tissues both in the epithelial plane and in 3D, and mechanical forces, together with cell shape and cell-cell adhesions, play an important role in controlling the orientation direction.

## 3 Mechanical forces shaping tissues during *Drosophila* morphogenesis

**Fig. 2:**
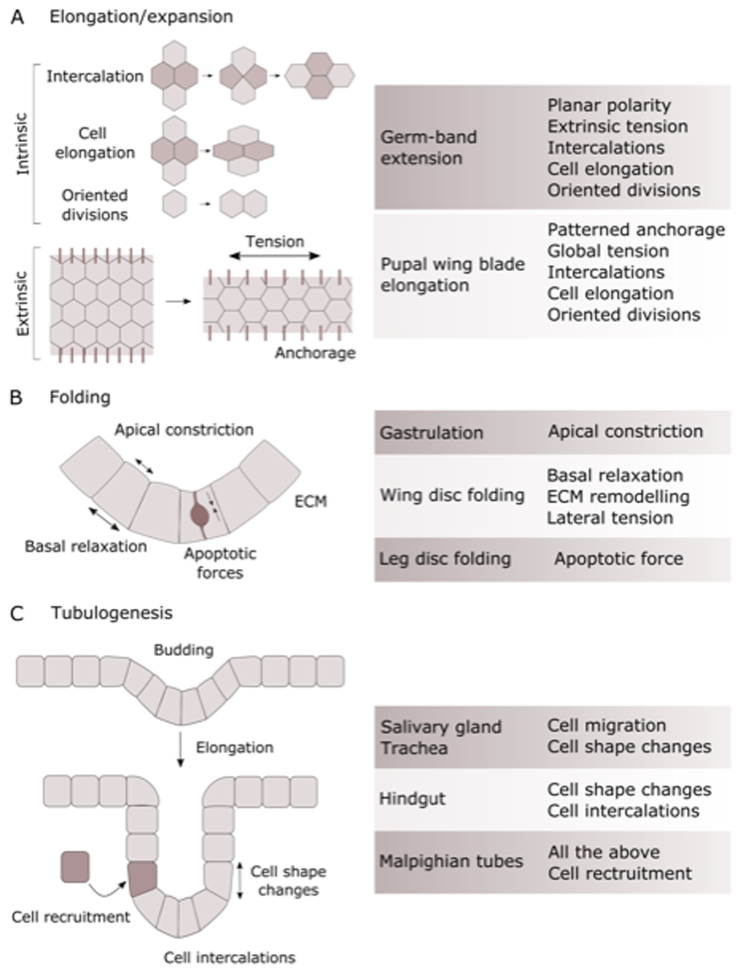
Morphogenetic processes in development. The figure shows key example of global tissue-sculpting processes driven by mechanical forces, with examples of their roles in the development of *Drosophila* organs. (A) Tissue elongation is a ubiquitous process that is driven by both intrinsic and extrinsic factors that cooperatively give rise to preferential elongation of the tissue along one axis. (B) Folding of epithelia is an essential process of 3D tissue shaping and it can be driven by different processes depending on the context (cell shape changes including apical constriction and basal relaxation, interactions with the ECM and the action of apoptotic forces). Note that while several mechanisms are shown on the figure these do not all occur in the same tissue. (C) Tube morphogenesis is key to the formation of several organs and it often initiates through a budding process, followed by elongation which can be achieved through different processes as illustrated.

Throughout *Drosophila* development, mechanical forces play an essential role in sculpting tissues into their functional 2D and 3D shapes. Cell autonomous and tissue-level forces have to be integrated in order to achieve the correct timing and execution of key morphogenetic movements, such as tissue elongation, folding and tube formation (Fig. 2).

### 3.1 Tissue elongation in the plane of the epithelium

*Drosophila* egg chambers are formed by a cluster of germline cells surrounded by an epithelial layer of follicle cells, which contacts a basement membrane (BM) on its basal surface. Egg chambers are initially round in shape but undergo a striking elongation along their anterior-posterior (AP) axis during maturation. Elongation is promoted by a global rotation of the egg chamber around the AP axis and coincides with the formation of a “molecular corset” of basal actin bundles in the cells and fibrils-like structures in the BM, which constrains growth of the egg chamber along the dorso-ventral axis [42], [43]. Recent work has shown that BM fibrils are generated *de novo* by secretion of newly synthetized proteins into the basal pericellular space, which are then inserted into the BM with a preferential orientation due to the tissue rotation [44].

Two classical examples of tissue elongation during *Drosophila* development are embryonic germ-band extension and pupal wing blade elongation. During germ-band extension, the posterior pole of the embryo undergoes invagination driven by Myosin II (MyoII) dependent apical constriction and the endoderm then moves towards the anterior. Polarized cell intercalations, driven by actomyosin planar polarization [45], [46] and by polarized basolateral protrusions [47], induce tissue elongation, as junctions in the anterior-posterior axis shrink selectively and new ones are formed between dorsal-ventral neighbors [45], [48], Fig. 2A. Recent work has revealed that the movement of the morphogenetic wave is guided by a tissue-scale wave of MyoII activation and subsequent cell invagination that establishes a mechano-chemical relay [49]. Extrinsic forces generated by concomitant morphogenetic movements also play a role in the process. Cell shape changes (antero-posterior elongation) have been shown to contribute to germ-band extension and arise from a global AP tensile force [50], which is in turn generated by apical constriction of the posterior endoderm primordium prior to endoderm invagination [51]. Finally, oriented cell divisions have also been shown to contribute to tissue elongation in the embryo [52].

Another striking example of tissue elongation that shared many similarities to germ-band extension occurs during pupal wing morphogenesis, when the wing blade is shaped by anisotropic tissue flows that induce its elongation in the PD axis and narrowing in the AP axis. These tissue flows are induced by a global tension pattern generated by hinge contraction and the anchoring of the wing margin to the pupal cuticle by the apical ECM protein Dumpy [53], [54]. Tissue shaping under this global tension pattern occurs through the concomitant action of cell shape changes, cell intercalations and oriented cell divisions [53], [54].

Overall, we see different mechanisms involved in the global elongation of tissues during development, often combining extrinsic constraints and intrinsic polarized intercalations, cell shape changes and oriented divisions (Fig. 2A).

### 3.2 Folding

Folding of epithelial sheets is a ubiquitous tissue shaping process in development, and folds of similar appearance can be generated by different mechanisms, with multiple force-generating elements often acting in parallel [55], Fig. 2B.

Cell-autonomous and tissue-level forces are often integrated to achieve folding at the tissue or even whole-embryo level. One famous example of such a process is gastrulation, where a uniform single-layer blastoderm undergoes a series of cell shape changes and movement to give rise to three distinct germ layers (the ectoderm, mesoderm and endoderm). The first step of gastrulation is ventral furrow formation, which is initiated by the expression of mesoderm transcription factors Twist and Snail, inducing accumulation of a MyoII network in the apical region [56], [57], [58]. Apical constriction of ventral furrow cells is generated by pulsatile contractions of the sarcomere-like actomyosin network, alternated to pauses in which the constricted state is stabilized, giving rise to an incremental constriction via a ratchet-like mechanism [59], [60]. Apical constriction induces a cell shape change from columnar to wedge-like, which also requires basal relaxation [61]. Due to global tissue tension being mostly directed along the furrow (AP axis), apical constriction is anisotropic, resulting in a long, narrow ventral furrow [62]. This polarized tension and its underlying actomyosin fibers orientation are organized by mechanical constraints imposed by global tissue shape and geometry, namely the rectangular shape of the ventral furrow region [63]. Despite the forces being locally generated in the prospective mesoderm, other parts of the embryo also participate in gastrulation and are required for mesoderm invagination. ﻿Cell populations along the dorso-ventral (DV) axis have been shown to respond differently to invagination of the ventral furrow, reflecting differences in cytoskeletal organization [64]. These mechanical heterogeneities control the transmission and coordination of forces to give rise to correctly timed dorsal widening, lateral cell displacement and furrow depth.

Folding of imaginal discs during the larval stage is often important to organize tissues in different domains. The *Drosophila* wing disc, for example, develops initially as a flat epithelium but acquires three stereotypic (major) folds within the prospective hinge region. These include the H/N fold (separating the prospective hinge and notum territories), the H/P fold (separating the prospective hinge and pouch territories), and a central H/H fold. This system is a great example of how, at the cellular level, different mechanisms can contribute to folding even within the same tissue. In fact, folding of the H/H fold was shown to occur through relaxation of the basal area of cells in the fold region, which requires ECM remodeling [65]. Formation of the H/P fold, on the other hand, occurs through a different mechanism involving increased lateral tension and subsequent pulsatile cell contractions that induce shortening of the cells on their apical sides [65]. Thus, cell shape changes into a wedge-like shape and the corresponding tissue sculpting are not exclusively driven by apical constriction.

These cell-autonomous forces also interact with tissue-wide forces, which can arise due to differential growth: in the developing wing disc, growth rates vary across the tissue, generating a tension pattern that is essential to ensure precise positioning of folds [66]. Finite element simulations incorporating experimentally-measured growth rates could correctly predict fold number and position, both in wild-type (WT) and mutant wing discs, and highlighted the importance of the BM constraining the tissue on the basal side to achieve fold initiation [66].

Another example of patterned folding occurs during the morphogenesis of the leg imaginal disc, where folds are formed at the location of presumptive joints between leg segments. This process has been shown to depend on local apoptosis, which is triggered by the activation of the pro-apoptotic gene *reaper* in a pattern of precisely located concentric rings [67]. Apoptotic cells undergo apical constriction and exert a pulling force on the apical side of the epithelium through a dynamic apico-basal actomyosin cable, deforming the surrounding tissue and inducing formation of folds [68]. Transmission of the apico-basal pulling force is enabled by the formation of a MyoII cable that connects the apical surface to the apoptotic nucleus, which is relocalized basally and anchored by F-actin to the basal side [69]. Contraction of the cable deforms the apical surface, transmitting force to the neighbors.

### 3.3 Complex 3D tissue sculpting

The fundamental processes that are involved in the elongation and folding of tissues can also shape tissues into more complex 3D structures. For example, epithelial tubes are a key component of several vertebrate and invertebrate organs (e.g. lung, vascular system, kidney) and undergo complex morphogenesis in order to form, elongate and branch into 3D structures [70], Fig. 2C. In *Drosophila*, two of the most well-characterized systems for tube morphogenesis are the salivary gland and trachea, which form from a polarized epithelium and are specified by patterning genes [71]. The first step of salivary gland tubulogenesis shares many similarities to tissue folding and occurs through apical constriction and internalization of the salivary primordia cells [72], [73], which no longer divide or undergo cell death after specification. Recent work has also highlighted the importance of oriented cell intercalations in the area surrounding the invaginating pit to achieve circumferential convergence and extension of the tissue towards the invagination center [74]. Similarly to salivary glands, tracheal invagination initiates through apical constriction of a small group of cells [75]. Throughout the internalization process, cell rearrangements and oriented mitotic divisions are also required to achieve the final tissue shape [76], [77]. Morphogenesis of the heart tube occurs through a different mechanism: the cardiac precursor cells migrate as two rows of cells towards the midline and they undergo shape changes, eventually joining with each other dorsally and then ventrally to close the tube and form the lumen [78]. Finally, the dorsal appendages of the *Drosophila* eggshell form through a “wrapping” tubulogenesis, where ﻿part of the epithelial sheet curls until its edges meet, sealing itself off and forming a tube parallel to the original epithelium [79].

Once they are formed, epithelial tubes elongate through different mechanisms, including ﻿changes in cell shape, cell rearrangements, cell division, and cell recruitment. In the salivary glands and trachea, tube growth is achieved through cell migration and cell shape changes [72], [80], [81]. In primary tracheal branches, ﻿cell rearrangements also contribute to elongation through a process that has been dubbed “stalk cell intercalation” [82]. As cells on the branch tip migrate, they induce a tensile stress in the tracheal branch which induces cell intercalations and further promotes tube elongation [82]. The *Drosophila* hindgut elongates in absence of cell division and apoptosis, through ﻿changes in cell shape (﻿from columnar to cuboidal), an increase in cell size and cell rearrangements [83]. Cell intercalations are oriented circumferentially due to a ﻿gradient of JAK/STAT pathway activation [84], which is essential to achieve proper elongation. The elongation of renal (Malpighian) tubules exploits a combination of all these mechanisms to give rise to an extended U-shape morphology [85]. In this system, additionally, cell recruitment occurs during elongation: mesenchymal cells are recruited to the tubules from the caudal visceral mesoderm and integrate into the epithelial tubules, where they differentiate into stellate cells [86], [87].

Another example of a global 3D remodeling process is the eversion of larval imaginal discs, which can be considered as an extreme case of tissue folding/unfolding, involving both the columnar disc proper and the squamous peripodial epithelia. Understanding of the coordinated movements that occur during eversion have been greatly aided by the development of long-term *ex-vivo* culturing and imaging of wing discs [88]. During eversion, the wing pouch protrudes and bends, bringing the dorsal and ventral compartments in apposition, while the peripodial epithelium initially expands to cover the larger surface created. The peripodial layer then retracts, ultimately forming a mass of rounded cells which are in large part eliminated through apoptosis [88], [89]. The global tissue remodeling that occurs during eversion also requires degradation of the wing disc BM by matrix metalloproteinases [90]. Eversion of the leg disc proceeds through similar steps of peripodial layer elongation, opening and removal; however, the BM contributes differently to the process [91]. During elongation of the peripodial layer, the BM and the cell layer become progressively uncoupled and the cellular monolayer later opens and withdraws independently of BM degradation, driven by myosin II-dependent contraction [91].

## 4 Mechanical pattern formation and refinement

In addition to global changes in size and shape, tissues also undergo patterning and pattern refinement during development, which may involve changes in cell arrangement, shape and size.

### 4.1 Complex 2D cell shape patterns

The morphogenesis of highly specialized organs often requires different cells to undergo distinct but coordinated developmental programs to achieve a functional tissue. A classic example is the *Drosophila* retina, which is composed of 750 units called ommatidia, each one ﻿comprising different cell types: cone cells, primary pigment cells and a ring of interommatidial cells [92]. These different cells are arranged in a complex yet precise pattern, with distinct packing and cell shapes, and this is essential for the function of the visual apparatus. Early observations that the arrangement of cone cells is reminiscent of soap bubbles [93] ﻿suggested that their overall shape might be optimized to minimize their contact surface, and differential expression of E- and N-cadherins was proposed to control pattern formation [93]. Subsequent work has shown that MyoII-dependent contractility is the main driver of cone cell shape [94] and that MyoII accumulation is in turn regulated by N-cadherin. More recent work has shown that a slow intercalation process that occurs between the four cone cells is largely independent of local MyoII activity, but instead relies of Neph/Nephrin-like adhesion and pulling forces external to the intercalating cone cells [95], highlighting the complex interplay between contractility and adhesion in pattern formation. Additionally, the specific mechanical properties of the different cells types have also been shown to be essential in the regulation of retinal morphogenesis [96]. All cell types have ﻿contractile medial MyoII meshworks that regulate their area and shape and enable mechanical coupling with each other; however, despite this mechanical coupling, cone cells are not deformed in response to forces because they are intrinsically stiffer than their neighbors [96]. Complex tissues as the fly retina might exploit differential cell stiffnesses to avoid ﻿averaging out of forces across cells during morphogenesis, which would normally prevent the acquisition of distinct apical geometries.

### 4.2 Compartment boundaries

The establishment of compartment boundaries during development ensures that cells with different fates remain segregated in order to achieve a correct tissue organization. The *Drosophila* wing disc is one of the most well-characterized tissues, organized in anterior/posterior (A/P) and dorsal/ventral (D/V) compartments which are established by the action of selector genes and signaling pathways [97]. Several studies have shown that the straight and smooth morphology of the D/V boundary is due to an increase in F-actin and MyoII during development [98], [99], which requires Apterous and Notch activity [100] and creates mechanical tension at the boundary that prevents mixing of adjacent cell populations. Similar evidence of F-actin/MyoII enrichment and increased mechanical tension has also been found at the wing disc A/P boundary [101], as well as embryonic parasegments boundaries [102]. Overall, these studies show that ﻿local increases in actomyosin-based mechanical tension on cell bonds are an essential mechanism to maintain compartment integrity during development. This can act in parallel to additional mechanisms, for example the differential expression of adhesion molecules between different compartments. One such example is the A/P boundary of pupal histoblasts, where a sharp expression boundary of the transmembrane receptor protein Toll-1 reinforces adhesion of homotypic cells, straightening the compartment boundary [103].

### 4.3 Epithelial packing and refinement

Planar cell polarity (PCP) plays a key role in the achievement of well-ordered cell packing during development, for example in the formation of ﻿hexagonally packed hairs on *Drosophila* wings, which have been suggested to affect airflow during flight [104]. PCP is established and regulated by the anisotropic distribution of key PCP proteins, including Frizzled and Disheveled (for a review see [105], [106]). Larval wing discs already display a global PCP pattern, but cell packing is irregular throughout larval and prepupal development [107]. In order to achieve the final global alignment of PCP domains with the proximal-distal (PD) axis of the wing, PCP and global tissue mechanics cooperate during the pupal stage [108]. More specifically, hinge contraction induces anisotropic tension in the PD axis and results in precise patterns of oriented cell elongation, cell rearrangement and cell division that elongate the blade proximo-distally and realign planar polarity with the PD axis [108].

Another example of packing refinement that is influenced by tissue mechanics is junction remodeling of the *Drosophila* pupal notum, which occurs in absence of global tissue deformations. In this system, stochastic fluctuations in junction length have been shown to give rise to numerous randomly oriented intercalations [109]. Over the course of development, the rate of intercalations gradually slows down as junctional MyoII levels increase isotropically, causing the tissue to become more ordered [109]. Overall this shows how global tissue mechanics, together with PCP, allow refinement of epithelial packing during development in order to achieve highly reproducible patterns of cellular organization.

## 5 Forces in homeostasis

Once a tissue has acquired its target size and shape, it will still retain a degree of plasticity that enables it to remodel and adapt in response to changes in the environment, while maintaining its overall size and shape. Several mechanisms contribute to this plasticity, which is essential for homeostasis and for the tissue to cope with internal and external mechanical insults, including wound healing.

### 5.1 Cellular processes driving plasticity of epithelia

Active cellular processes important in tissue homeostasis include cell divisions, intercalations and extrusions, all of which can contribute to stress dissipation. As we have previously discussed in the context of tissue growth during development, cell divisions can orient in response to tension [24], [25] and this could help restore the epithelium homeostatic state [110], [111]. In addition, we have seen that mechanical forces can influence local growth rates to help tissues achieve and maintain their final size, promoting local growth to relieve tension and inhibiting growth in crowded conditions [7], [8], [9].

**Fig. 3:**
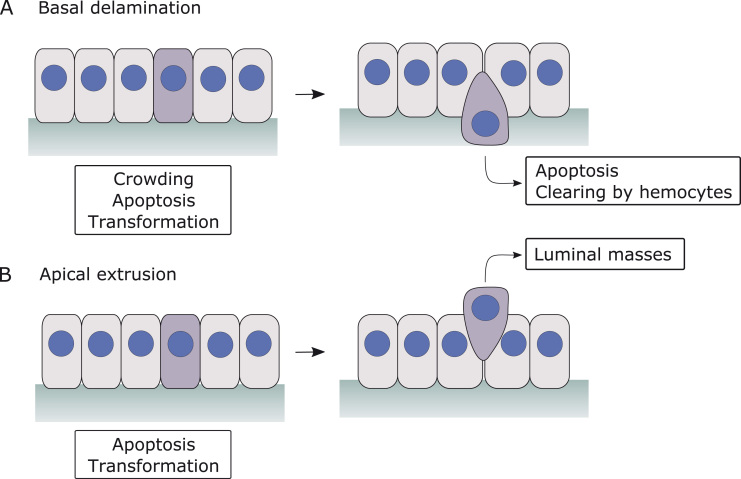
Cell elimination from epithelia during development and homeostasis. The figure illustrates the two main processes by which cells can be eliminated from an epithelium: basal delamination (A) and apical extrusion (B). In *Drosophila*, basal delamination is the most common process and it results in apoptosis of the delaminated or clearing by haemocytes. A few examples of apical extrusions have also been reported in apoptotic or transformed cells, a process which can cause the accumulation of luminal masses.

In conditions of overcrowding, however, an additional mechanism can support stress dissipation: cells can be eliminated from the epithelium by extrusion (at the apical surface) or delamination (at the basal surface) [112], [113]. Basal delamination is by far the most common process in *Drosophila*, and it typically results in apoptosis or clearing of the cell by haemocytes, Fig. 3A. Examples of apical extrusion are found in the elimination of apoptotic enterocytes in the adult midgut [114] and in some tumor models [115], where the extruded cells can form luminal masses [116], Fig. 3B. In the *Drosophila* notum, cell delamination was shown to occur randomly in areas experiencing cell crowding, a potential mechanism to ensure the formation and maintenance of a regular cell packing and relieve mechanical stresses in the tissue [117]. Recent studies found that caspase activation is involved in crowding-induced delamination in the notum [118] and that it is triggered by transient changes in EGFR/ERK signaling following tissue stretching or compaction [119].

These local interactions can be put into the broader context of cell competition, a process whereby “loser” cells characterized by a lower fitness are gradually eliminated from a tissue [120]. On the other hand, even WT cells can be outcompeted by mutations that increase the fitness of cells and turn them into “winners”, in a process often termed “super-competition” [121]. Cell competition has been found to occur through several processes including the competition for survival factors, the display of fitness markers identify less fit cells and, more recently, mechanical competition between neighboring cells, in which winner cells compress their neighbors, increasing the local cell density and promoting the elimination of loser cells (see [122] for a review). The compression-driven cell extrusions found in the *Drosophila* notum and described earlier represents a perfect example of the latter type [117], [118], [119]. Importantly, mechanical competition is likely involved in tumorigenesis: clones of cells with active oncogenic Ras^V12^, for example, are more resistant to compaction than neighboring cells, giving rise to clone expansion [118].

Overall, by responding to mechanical cues such as tension and crowding, developed epithelia can maintain a level of plasticity which enables them to adapt to changes in the environment and achieve homeostasis.

### 5.2 Tissue-level response to mechanical forces

The cellular-level processes enabling tissues to adapt to mechanical forces described in the previous section can only act over relatively long periods of time, on a timescale of minutes to hours [111]. How do tissues respond to and buffer forces on shorter timescales? The most immediate response to an applied force will depend on the material properties of the tissue (e.g. its elasticity). In addition to this, recent work on *Drosophila* wing discs has shown that epithelia can also rapidly remodel to adapt to sudden mechanical perturbations [123]. Stretching wing discs induces the rapid formation of supracellular actomyosin cables that globally stiffen the epithelium, constraining cell shape changes and preventing the propagation of fractures. Cable formation upon stretch is dependent on the actin nucleator Diaphanous, which is also responsible for F-actin remodeling that allows the tissue to gradually dissipate tension over time [123]. Overall, this shows how epithelia can buffer mechanical stresses across different timescales by exploiting both cellular and tissue-scale processes.

### 5.3 Wound healing

Another key homeostatic process in epithelia is their ability to heal wounds and maintain integrity after a tissue damage. The importance of mechanical factors in wound healing has been highlighted by several studies both *in vitro* and *in vivo* [124], with *Drosophila* representing a widely employed model system [125].

When *Drosophila* embryos are wounded (either mechanically or with laser ablations), an actin cable forms at the wound edge, operating as a purse-string to close the wound, similar to the machinery involved in dorsal closure [126], [127]. Dynamic filopodia also form in leading edge cells, which make contact with each other in the final stages enabling wound sealing. In the absence of an actin cable, neighboring lamellipodia can tug on each other and still enable a complete (albeit slower) wound closure [126]. Cell shape changes also play a role in wound closure: cells spanning several rows away from the wound edge, in fact, have been observed to elongate towards the wound [128]. Additionally, cells positioned anterior-posterior to the wound extend in width to contribute to wound closure and exhibit ratchet-like junction shrinking that gives rise to cell intercalations, reminiscent of the process occurring during germband extension [128]. The interplay of all these mechanisms confers flexibility to the wound repair machinery and enables wounds of different topology to be closed effectively (e.g. narrow incisional wounds can be rapidly closed by lamellipodia zippering only, whereas larger round wounds also require a purse-string mechanism [126]).

The mechanism of wound closure not only depends on wound morphology, but it can also differ in different tissues. In wounded larval wing discs, a purse string similar to the one found in embryos forms but no protrusion-based migration of the wound edge is observed. Two phases of wound healing are observed: an initial fast phase mostly driven by the purse string where wound area reduces by 50%, followed by a slow one that progressively leads to complete wound closure. The slow phase was found to depend on numerous intercalations of wound edge cells, that help preserve cell shape after an initial transient elongation of cells towards the wound [129]. Simulations confirmed that an increased tissue fluidity can compensate for a reduced purse string, however the wound cannot be completely closed by intercalations only [129]. This study highlights how the mechanical properties of the surrounding tissue can play a role in wound closure in addition to local wound-closure machinery.

Additional mechanisms that help alleviate the mechanical stress induced by wounding are polyploidization and cell-cell fusion [130], [131], [132]: these processes result in the formation of very large cells near the wound edge, which might ﻿allow the establishment of robust cytoskeletal structures and mechanically stabilize wounds. Compensatory proliferation is another response to injury that can enable impressive regeneration of damaged tissues [133]. Proliferation is often induced locally around the wound site and requires the action of several signaling pathways including Wingless, JAK/STAT and Hippo (see [134] for a review). Importantly, increased proliferation coordinates with ﻿changes in cell division orientation and cell fate re-specification to achieve regeneration of tissues with the correct size and shape [135].

In summary, tissues employ several mechanisms to heal wounds and maintain their integrity, including an actomyosin purse string, cell shape changes, cell migration, and tuning tissue fluidity (rate of intercalations). Often multiple mechanisms act in parallel, to guarantee robust and seamless wound closure.

## 6 Developmental robustness

Developmental patterns are strikingly reproducible and robust [136]. In recent years, several studies have looked into the role of tissue mechanical properties in developmental robustness.

In the *Drosophila* leg disc, a precise pattern of four parallel folds forms during development and perturbations of Arp2/3 complex component were found to induce deviated folds [137]. Planar polarization of MyoII was shown to be necessary to render the folds insensitive to mechanical perturbation: without it, folds initiate properly but propagate with low precision due to local mechanical noise (e.g. they deviate towards regions of high tension) [137]. The polarization of MyoII ensures that force transmission is biased in the direction of future fold formation, “buffering” the effect of additional forces in the tissue.

Supracellular structures were also found to be important to confer robustness to cephalic furrow (CF) formation in *Drosophila* embryos [138]. The CF is positioned with a very high precision (on the order of one cell diameter) in WT embryos [139]. Initiating cells are specified with single-cell resolution by the expression patterns of *btd* and *eve*, however this positional code was found to account only for 80% of the initiation events. Despite these inaccuracies in specification, CF initiation displays a precise spatial alignment which is ensured by tissue-scale mechanical coupling by supracellular myosin “ribbons” [138]. This is another example of how mechanical coupling can function as a noise correcting mechanism to ensure robust morphogenesis.

The structure and function of supracellular actomyosin networks during morphogenesis was recently investigated using a novel tracing method to monitor the network structure during *Drosophila* ventral furrow folding [140]. Interestingly, many more connections are found in WT actomyosin networks than are minimally required to fold the tissue: this redundancy thus provides one layer of robustness to the system in the face of potential disruptions. Furthermore, stiffening of network connections along the A-P axis promotes robust folding of the furrow along the correct axis.

As we have discussed in the previous sections, mechanosensitive polarization of MyoII can act as a rapid response to external forces to limit cell shape changes [123]. Such mechanosensitive response also plays a role during development, for example in the polarization of MyoII in response to forces caused by proliferation anisotropy in the wing disc [24], [25]. Supracellular cables circumferential around the wing pouch act as mechanical feedback to limit tissue deformation in response to global forces, conferring robustness to tissue shape [24], [25].

In addition to the above examples illustrating the importance of tissue-level mechanical organization in morphogenetic robustness, further proofreading mechanisms can act during development. For example, during the formation of the *Drosophila* cardiac vessel, two opposing rows of cardioblasts migrate to the central midline and have to precisely match with their contralateral partner cells, binding through filopodia [141]. Proofreading of these connections is achieved through MyoII oscillations, which periodically forms foci at the leading edge of the migrating cells, inducing the retraction of weakly connected filopodia and reinforcing strongly connecting filopodia to ensure robust matching between contralateral cardioblasts [141].

## 7 Conclusions and future perspectives

The different processes described above illustrate the central role of mechanical forces in *Drosophila* tissue morphogenesis and homeostasis. Despite the striking advancements in the last few years, our understanding of the physiological forces acting *in vivo* during processes such as morphogenesis or wound healing is still lacking as *in vivo* biophysical experiments remain challenging. It will be crucial to further develop imaging techniques capable of probing the mechanical properties of tissues *in vivo* without perturbing them, such as the newly emerging Brillouin microscopy [142]. These could be combined with methods that enable the local perturbation of forces *in situ*, such as the ever-growing optogenetic toolbox [143], as well as the use of injected ferrofluid droplets to both apply and monitor local forces *in vivo* [144], [145], [146]. The development of methods such as correlative light-sheet and AFM [147] that enable simultaneous imaging and force measurement is also very promising, together with continuous improvements of genetically encoded sensors to measure piconewton-range forces across individual molecules [148]. As exemplified by many studies discussed in this review, mathematical and computational models have also become powerful approaches in developmental mechanobiology [149], [150]. There is increasing interest in models integrating mechanical and signaling aspects [151], [152], extended up to three dimensions [153], as well as models incorporating stochastic elements [154], [155], [156], [157] which could help better understand the role of genetic and mechanical noise [158] in morphogenesis.

Looking ahead, these new technologies will be essential to address open questions in the field, and to integrate mechanical information in 3D. Through many examples discussed in this review, we have seen that tissues respond differently to mechanical forces at different developmental stages, and morphogenetic processes that ultimately give rise to a similar shape (e.g. fold, tube) can proceed through dramatically different steps. It will be important to further dissect the reasons behind these differences, both in terms of the molecular signals upstream and downstream of the tissue response and correlate these closely to the unique properties of the mechanical strains involved (magnitude, timing). Furthermore, it will be important to better understand how force buffering acts during homeostasis and repair to confer robustness to tissues against varying intrinsic and extrinsic patterns of mechanical forces. This might eventually shed light on how organisms evolved tissues of distinct shape and size to perform specific function in a mechanically-active and noisy environment.

## Conflict of interest

The authors declare no conflict of interest.
